# Skin of Color in Pediatric Dermatology: A Cross-Sectional Retrospective Analysis Addressing Inclusive Diagnosis and Care

**DOI:** 10.3390/life16040578

**Published:** 2026-04-01

**Authors:** Arianna Dondi, Alice Ranieri, Laura Andreozzi, Miriam Leuzzi, Gabriele D’Alanno, Luca Pierantoni, Daniele Zama, Eleonora Battelli, Roberta Calegari, Andrea Borghesi, Marcello Lanari, Iria Neri

**Affiliations:** 1 Department of Medical and Surgical Sciences, Alma Mater Studiorum, University of Bologna, 40138 Bologna, Italy; arianna.dondi2@unibo.it (A.D.); marcello.lanari@unibo.it (M.L.); 2 Pediatric Emergency Unit, IRCCS Azienda Ospedaliero-Universitaria di Bologna, 40138 Bologna, Italy; laura.andreozzi@aosp.bo.it (L.A.); luca.pierantoni@aosp.bo.it (L.P.); eleonora.battelli@aosp.bo.it (E.B.); 3 Specialty School of Paediatrics, Alma Mater Studiorum, University of Bologna, 40138 Bologna, Italy; alice.ranieri3@studio.unibo.it (A.R.); gabriele.dalanno@studio.unibo.it (G.D.); 4 Unit of Dermatology, IRCCS University Hospital of Bologna, Policlinico S. Orsola-Malpighi, 40138 Bologna, Italy; miriam.leuzzi2@unibo.it (M.L.); iria.neri@aosp.bo.it (I.N.); 5 Department of Computer Science and Engineering (DISI), Alma Mater Studiorum, University of Bologna, 40138 Bologna, Italy; roberta.calegari@unibo.it (R.C.); andrea.borghesi3@unibo.it (A.B.)

**Keywords:** dermatology, paediatrics, skin pigmentation, atopic dermatitis

## Abstract

Children with skin color (SOC) are underrepresented in dermatologic research, despite structural and functional differences that shape disease presentation. Atopic dermatitis (AD), one of the most common pediatric dermatoses, often appears differently in SOC than in white children. This study compared dermatologic conditions prompting Pediatric Emergency Department (PED) referral in SOC and white children, and described clinical features of AD in SOC. A retrospective study was performed at IRCCS AOUBO Policlinico di Sant’Orsola, Bologna, Italy, analyzing records and photographs from 2019. Patients presenting with dermatologic conditions and evaluated by a pediatric dermatologist were included. Of 411 patients, 109 (26.5%) had SOC. In SOC, common diagnoses were scabies (22%), AD (17.4%), viral infections (12.8%), burns (9.2%), and contact dermatitis (7.3%). In white children, viral infections (16.9%), burns (14.2%), contact dermatitis (13.9%), AD (12.9%), and insect bites (5.6%) predominated. Scabies and pruritus were significantly more frequent in SOC (*p* < 0.05). Among 38 SOC patients with AD, lichenoid (31.6%), pityriasis alba (29.0%), prurigo nodularis (26.3%), and classic AD (13.2%) were the most frequent variants. Erythema was often subtle or absent. Dermatologic conditions and AD morphology differ between SOC and white children, highlighting the need for tailored diagnostic approaches and equitable care.

## 1. Introduction

Skin of color (SOC) describes individuals with skin tones darker than those of Caucasians. The term primarily refers to phenotypic skin characteristics, typically corresponding to Fitzpatrick phototypes IV, V, or VI [1], which influence the clinical presentation of dermatologic conditions. However, ethnicity represents a broader sociocultural construct that may reflect differences in environmental exposures, socioeconomic determinants, healthcare access, and cultural practices. Diseases affecting SOC patients, including children, remain notably underrepresented in the literature [2,3]. Quantitative evidence further highlights this imbalance; an analysis of dermatologic images from widely used online resources found that only 11.7% represented melanin-rich skin, with more than 90% of searches demonstrating underrepresentation of SOC relative to population demographics [4]. This underrepresentation underscores the growing importance of considering ethnic diversity in medicine, particularly in dermatology. In Italy, the demographic composition is becoming increasingly diverse, with a rising proportion of patients with SOC. This trend provides a valuable opportunity to investigate dermatological conditions within this population.

SOC presents specific structural and functional characteristics involving, for instance, the stratum corneum, dermis, and melanin production. Darker skin has larger, more dispersed melanosomes rich in eumelanin, providing greater UV protection. Its stratum corneum is generally more compact but may show lower ceramide levels and increased water loss. In the dermis, larger and more numerous fibroblasts may contribute to a higher tendency for keloid formation. These features influence the clinical expression of skin diseases, meaning that dermatological conditions in patients with dark skin may differ substantially from those described in Caucasian populations. The lack of adequate documentation—particularly iconographic—of dermatological manifestations in SOC can hinder clinical recognition, resulting in delayed or inaccurate diagnoses. In addition, many dermatological teaching resources remain predominantly based on lighter skin types, which may contribute to diagnostic delays and disparities in care.

Atopic dermatitis (AD) is among the most studied dermatological disorders due to its considerable clinical heterogeneity across skin types. It is one of the most common chronic inflammatory diseases of childhood, affecting up to 20% of children [5]. AD is a chronic-relapsing condition that typically begins in early life and often remits during adolescence [6,7]. However, recent studies show that onset may vary widely, with some cases first emerging in adolescence or adulthood [8].

Descriptions of AD in standard textbooks are largely based on its presentation in Caucasian patients. In this population, classic AD usually begins before the age of two with itchy erythema and acute or subacute eczema, later evolving into subacute or chronic lichenified lesions. In non-Caucasian patients, however, AD often manifests with distinctive variants [9]. These include prurigo nodularis [10], characterized by hyperkeratotic and hyperpigmented nodules; pityriasis alba, presenting with multiple hypopigmented patches; the follicular or lichenoid variant [11], with papules clustered around follicular ostia; and nummular eczema, marked by rounded, scaly plaques [9], which is especially common in Asian populations [12]. Moreover, the classic age-related distribution of AD—extensor surfaces in early childhood and flexural surfaces in later stages [13]—is less consistent in SOC, where extensor involvement remains more frequent [14].

In children with SOC, erythema may be absent or appear as violaceous, purple, or brown hues that are difficult to assess, making inflammatory processes more difficult to recognize. Indirect signs such as edema, increased warmth, or scaling may serve as clues [10]. Xerosis also tends to be more pronounced, leading to presentations dominated by pruritus without obvious erythema, which complicates diagnosis [15]. Furthermore, AD in SOC patients is often more severe and more challenging to treat, highlighting the need for tailored management strategies [15]. Although AD represents one of the most frequently discussed inflammatory conditions in patients with skin of color, a broad spectrum of pediatric dermatoses may present differently in this population: common conditions such as viral exanthems, urticaria, scabies, pediculosis, and drug-related eruptions may display variations in erythema visibility, pigmentation changes, or lesion morphology when occurring in darker skin phototypes. These differences can complicate clinical recognition and may contribute to delayed diagnosis or misclassification in children with SOC.

The primary aim of this study is to investigate distribution of dermatological discharge diagnoses among Pediatric Emergency Department (PED) visits evaluated by a pediatric dermatology consultant in children with SOC compared with white children. The secondary aim is to describe the specific clinical features of AD in pediatric SOC patients, focusing on morphology and lesion distribution, and to compare these findings with those reported in Caucasian patients.

## 2. Material and Methods

We conducted an observational, retrospective, monocentric study by reviewing the electronic medical records and pictures of pediatric patients with SOC who accessed the Pediatric Dermatology Outpatients’ Service of IRCCS AOUBO Policlinico di Sant’Orsola in Bologna, Italy, either by direct access or after an evaluation in the PED for a dermatologic condition, from 1 January to 31 December 2019. The study was approved by the local Ethics Committee Area Vasta Emilia Centro (AVEC, protocol number 341/2023/Oss/AOUBo).

SOC was defined as Fitzpatrick skin phototypes IV to VI, as previously reported [16]. The study consisted of two complementary analyses: (1) evaluation of dermatological diagnoses among pediatric patients presenting to the PED and (2) description of morphological variants of atopic dermatitis in SOC patients evaluated in the dermatology outpatient clinic.

In order to investigate the primary aim of the study, all pediatric patients (aged 0–14 years) who accessed the PED with a dermatologic condition were selected through a data extraction method that could extract specific dermatologic strings from the patients’ electronic records in the “clinical examination” and “discharge diagnosis” fields. This method was preferred over the classic selection according to the ICD classification system to include those patients who accessed the PED for other conditions but also had a dermatologic issue. Patients who were NOT evaluated by the Pediatric Dermatology Outpatients’ Clinic consultant were excluded. The upper age limit of 14 years reflects the organizational structure of our Pediatric Emergency Department, which provides care for patients up to this age.

Finally, patients were classified as having white skin or SOC as documented in the medical record or through evaluation of available pictures; patients of Asian ethnicity were excluded, because their number in the cohort was insufficient to allow meaningful statistical analysis. The final PED discharge diagnosis was recorded. The diagnoses were divided into 18 categories: scabies, entomodermatoses other than scabies, viral infections, bacterial infections, mycosis, urticaria, burns, atopic dermatitis, seborrheic dermatitis, contact dermatitis, purpura, vascular lesions, itching, psoriasis, lichen, drug reaction and others (i.e., neonatal cephalic pustulosis, epidermal cyst, dyschromia, hyperkeratotic plaque, scalp nodules, dermatomyositis). Additional methodological details are provided in Supplementary File S1.

Categorical variables were described as frequencies and percentages, whereas continuous variables were described as the median and interquartile range due to the non-normal distribution of the data. Normality was evaluated using the Shapiro–Wilk test, as well as graphical methods (histograms and Q–Q plots). Categorical variables were compared by the Chi-square test or Fisher’s exact test, as appropriate. When the overall Chi-square test or Fisher’s exact test was significant, post hoc pairwise comparisons were performed using Fisher’s exact tests. To control multiple comparisons, Bonferroni correction was applied to the *p*-values.

For the secondary aim of the study, all patients with SOC who performed a dermatologic evaluation for AD at the Pediatric Dermatology Outpatients’ Clinic during the study period and for whom pictures of the skin condition were available, were included. We collected the patients’ demographic data including age, gender, country of origin, and citizenship. Only patients with SOC, as documented in the medical record or through evaluation of the pictures, were included. Two pediatric dermatologists (IN and ML) independently reviewed the pictures in order to define the clinical features of AD. In case of discordance, the case was discussed to find an agreement. Subsequently, for each patient the variant of AD (classic [14], prurigo nodularis [10], pityriasis alba and follicular or lichenoid variant [11], and nummular eczema [9]) and the anatomical distribution of the lesions were recorded and compared to existing classifications [6]. Each patient was assigned a predominant morphological variant based on the dermatologists’ evaluation of the available images. Descriptive statistics, including frequencies and IQR, were performed for the secondary aim.

## 3. Results

(a) Dermatological conditions referred to the PED in children with SOC or white skin

**Figure 1:**
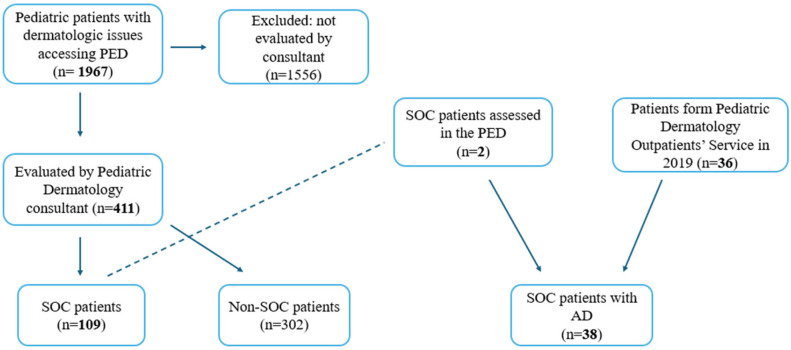
Flow diagram of patient selection and cohort inclusion.

During the study period, 1967 pediatric patients with a dermatological issue accessed our PED. Among them, 411 (20.8%) were evaluated by the Pediatric Dermatology Outpatients’ consultant and were included in the analysis; of them, 109 (26.5%) patients had SOC. The patient selection process is summarized in Figure 1.

The median age at first visit was 4.36 years (IQR 1.51–8.65); 201 were female (48.9%) and 210 male (51.1%) patients.

**Figure 2:**
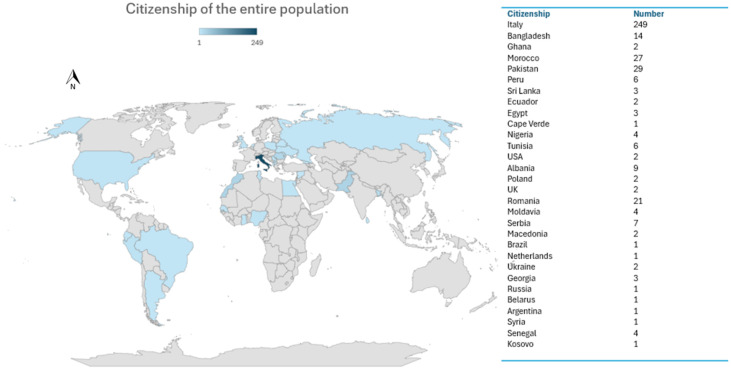
Citizenship of 411 patients who accessed the Pediatric Emergency Department (PED) of IRCCS AOU di Bologna during 2019 for dermatological issues and were evaluated by a pediatric dermatologist.

The demographic characteristics of the patients are reported in Figure 2.

### Dermatological Diagnoses at Discharge

Discharge diagnoses in patients with SOC and with white skin are reported in Table 1. The top five common diagnoses found in all pediatric patients were viral infections (15.8%), AD (14.1%) and burns (12.9%). Particularly, if a focus on the first five discharge diagnoses in white patients highlighted viral etiology (16.9%), burns (14.2%), contact dermatitis (13.9%), AD (12.9%), and insect bites (5.6%), in SOC patients were scabies (22%), AD (17.4%), viral infections (12.8%), burns (9.2%), contact dermatitis (7.3%).

The post hoc analysis compared the relative frequencies of various dermatological diagnoses between white and SOC populations and showed that diagnoses of scabies and itching are significantly more present in SOC patients. Scabies was substantially more prevalent among SOC patients compared to other skin conditions, such as entomodermatoses, viral infections, bacterial infections, mycoses, urticaria, burns, contact dermatitis, AD, drug reactions, vascular skin diseases, and other conditions. Itching was significantly more frequent in SOC patients compared to viral infections, bacterial infections, urticaria, burns, contact dermatitis, vascular diseases, other conditions.

No meaningful differences were found for seborrheic dermatitis, psoriasis, acne, lichen planus, purpura.

Results of the post hoc analysis are shown in Supplementary File S1.

(b) Clinical features of AD in pediatric patients with SOC

A cohort of 38 patients with SOC was enrolled, including 2 patients assessed in the PED and 36 additional patients evaluated at the Pediatric Dermatology Outpatients’ Service in 2019 for whom iconographic documentation was available. Demographic characteristics of the population with AD who underwent dermatologic examination and description of the morphologic variants are shown in Table 2.

The most common variant of AD was lichenoid (12; 31.6%), followed by pityriasis alba (11; 29.0%), prurigo nodularis (10; 26.3%), and classical (5; 13.2%). The affected body locations were recorded, revealing typical age-related sites in 66% of cases and the extensor surfaces (i.e., wrists, elbows, ankles, and knees) in 44%. Lesion distribution categories were not mutually exclusive, and some patients presented involvement of both typical age-related sites and extensor surfaces. Specifically, these extensor locations were involved in 100% of the cases of the follicular variant of AD.

## 4. Discussion

In our urban Italian setting, clear differences emerged in the dermatological conditions leading to referral to the PED between children with SOC and those with white skin. Among SOC children, the most frequent discharge diagnoses were scabies and AD, whereas referrals of white children were more often related to viral exanthems and burns. The confirmation of diagnoses by a pediatric dermatologist strengthens the diagnostic reliability and clinical significance of our findings.

These results highlight the importance of recognizing how dermatological presentations may vary not only in morphology but also in epidemiological distribution across different skin phototypes. The higher frequency of AD among SOC patients compared with Caucasians is consistent with previous studies [17]. The greater frequency of scabies and AD in SOC populations may reflect socioeconomic, environmental, and structural determinants, as well as diagnostic delays due to challenges in detecting skin conditions in darker phototypes. The higher frequency of scabies should not be interpreted as reflecting biological susceptibility, rather differences in exposure dynamics, household transmission, and structural determinants such as crowding or access to healthcare. Conversely, the higher incidence of viral exanthems and burns among white children may be linked to different exposure patterns, health-seeking behaviors, or access to preventive measures.

Such variation underlines the need for cultural and dermatologically competent care in diverse pediatric populations. It also emphasizes the necessity of improved training for pediatricians and emergency physicians in recognizing skin diseases across all phototypes. Further research is required to clarify the underlying causes of these disparities and to guide targeted clinical strategies.

The high proportion of itching in SOC patients is consistent with the increased frequency of scabies, in which pruritus represents the most common feature. It may also reflect underdiagnosed or undertreated conditions such as AD. Recent European surveillance reports confirm rising scabies frequency, especially among vulnerable groups [18]. Both scabies and AD share pruritus, often nocturnal, with relief from cold and worsening with heat. Itching in both disorders is mediated by a T-helper 2 immune response, involving pruritogenic cytokines (IL-4, IL-5, IL-13, IL-31) and their receptors on cutaneous sensory neurons [19]. Therefore, in SOC children presenting with itching, careful family history (atopy or parasitic infestations) and thorough physical examination are essential. White patients in our study more often presented with allergic, inflammatory, infectious, and iatrogenic conditions, possibly reflecting greater access to dermatological care and earlier detection of non-parasitic diseases.

The “classic” AD morphology—erythematous, scaly plaques in flexural sites—is described mainly in Caucasian populations [20]. In contrast, “atypical” variants are more common in SOC, including prurigo nodularis, pityriasis alba, follicular/lichenoid forms, and nummular eczema [9,18], with extensor involvement occurring more frequently^13^. Erythema in SOC may appear violaceous, brown, or grayish, or be suggested only by indirect signs such as edema, warmth, or scaling [10,21]. These differences may contribute to diagnostic delays or underdiagnosis in children with darker skin.

**Figure 3:**
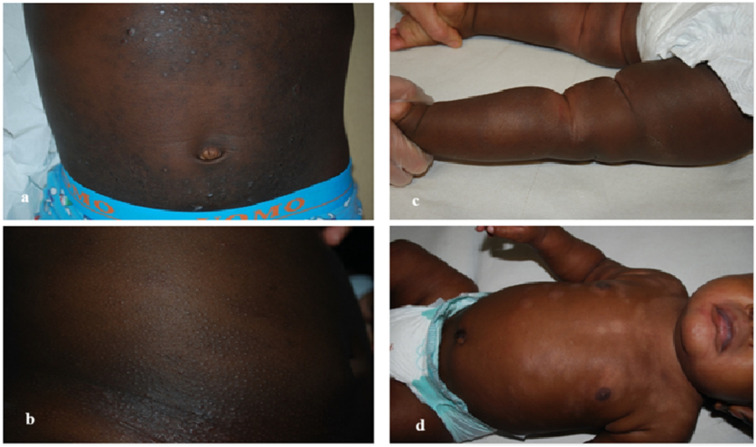
(**a**,**b**) **Follicular or lichenoid variant**. Characterized by papules surrounding the follicular openings, often found on the extensor surfaces. (**c**,**d**) **Pityriasis eczematid variant**. Round or oval hypopigmented patches covered with thin pityriasis scales.

**Figure 4:**
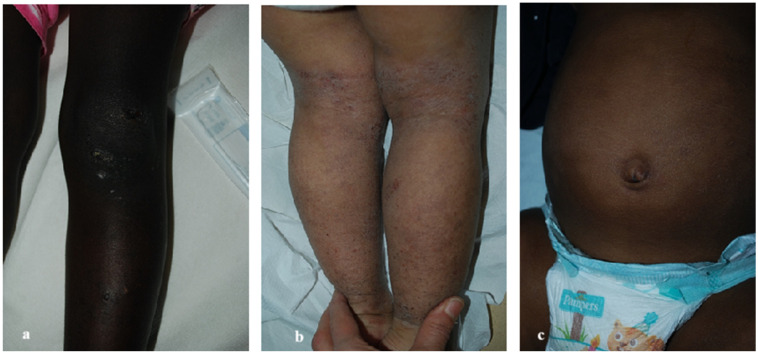
(**a**) **Prurigo eczema.** Defined by itchy nodules which tend to be hyperkeratotic, hyperpigmented and excoriated. (**b**,**c**) **Erythematous-desquamative variant.** Erythema in SOC patients tends to occur in purplish, grayish or dark brown shades rather than red.

In our cohort, the most frequently observed variants of AD were follicular or lichenoid (Figure 3a,b), though less prevalent than the ~55% reported in other studies [22,23]. Pityriasis alba (Figure 3c,d) affected 29% of our patients, ranking second in frequency, compared to ~50% proportion in SOC and ~15% in non-SOC populations [24]. Prurigo nodularis (Figure 4a) accounted for ~26% of cases, close to Herzum et al.’s 32% in SOC patients and substantially higher than the ~5% in Caucasians [24]. The classic erythematous-desquamative variant (Figure 4b) was found in 13.2% of cases, significantly lower than the up to 95% frequency described in non-SOC populations [25]. Importantly, erythema in SOC often appears violet, grayish, or dark brown rather than red [9,21].

In clinical practice, the assessment of AD severity in SOC may require greater attention to additional features beyond erythema, including papulation, lichenification, excoriations, dyspigmentation patterns, and patient-reported symptoms such as pruritus and sleep disturbance. This creates significant challenges in diagnosis and in assessing disease severity, as widely used scoring tools (SCORAD, EASI) rely heavily on erythema evaluation [26]. Their reduced reliability in SOC populations may compromise severity assessment and limit access to systemic therapies [27]. Literature also indicates that SOC patients tend to experience more severe disease courses than Caucasians [28], and disparities in outpatient access for eczema care [29] may further exacerbate this gap. In our sample, no cases of the nummular variant were found, consistent with reports describing it primarily in Asian populations [12].

Regarding lesion distribution, most patients (66%) presented with age-appropriate patterns. However, nearly half (48.3%) of those in later childhood and adolescence had prominent extensor involvement, supporting literature reports that SOC patients maintain extensor involvement beyond early childhood [10,14,23,30], unlike non-SOC populations, where extensor surfaces are usually spared [21,31].

Emerging artificial intelligence tools may further support dermatologic diagnosis across diverse skin phototypes. Recent studies have shown promising performance of large language model-based image recognition systems in dermatology [32,33]. Furthermore, studies have highlighted how artificial intelligence applications in dermatology may reproduce existing biases due to limited representation of darker skin tones in available datasets [34]. In the future, similar approaches could be applied to pediatric dermatology workflows, using clinical photographs from emergency department and outpatient documentation to support standardized morphological classification and differential diagnosis in SOC populations.

The present study was designed as a retrospective, single-center analysis to provide preliminary data on dermatological conditions and AD presentations in pediatric patients with SOC within our local context. SOC status was determined from medical record documentation and, when available, from clinical photographs reviewed by pediatric dermatologists; however, given the retrospective design and variability in photographic documentation, some degree of misclassification cannot be excluded; furthermore, these findings derive from a single tertiary pediatric emergency department in an urban Italian setting and may not be generalizable to other healthcare systems or populations. Despite these limitations, this approach enabled a systematic review of clinical and iconographic records, allowing an initial characterization of dermatological diagnoses and morphological variants of AD in this underrepresented population. Another possible limitation is the lack of data on key social determinants of health—such as socioeconomic status, ethnic lifestyle and upbringing, and healthcare adherence—which may influence both the epidemiology and the clinical presentation of dermatological conditions in pediatric patients with SOC. The analysis included only patients assessed by a dermatologist, and therefore the ethnic distribution of non-assessed patients could not be evaluated. Our group is currently conducting a prospective study on dermatologic conditions in patients with SOC that incorporates several social determinants of health; however, additional time will be required to accrue a sufficiently large dataset for a more comprehensive characterization of these conditions.

## 5. Conclusions

Overall, dermatological descriptions of SOC patients, particularly children, remain underrepresented in the literature. Our findings contribute new data on referral diagnoses and morphological variants of AD in pediatric SOC populations, aligning with previous studies. We confirm a high frequency of follicular/lichenoid variants, pityriasis alba, and prurigo nodularis, while the classic variant remains less frequent than in Caucasian populations. Although age-related distribution was typical in most cases, extensor involvement was notably frequent in SOC patients.

As the ethnic composition of Western countries continues to evolve, improved recognition of dermatologic conditions in SOC populations is essential to ensure equitable and accurate care. Differences in AD morphology and distribution exist between SOC and white patients highlight the limitations of diagnostic approaches historically derived from Eurocentric skin types. Pediatricians and pediatric dermatologists must be aware of these differences to ensure timely and accurate diagnosis, appropriate treatment, and optimal prognostic outcomes [35]. From a practical perspective, educational initiatives incorporating dermatologic images across diverse skin phototypes should be integrated into pediatric and emergency medicine training, and healthcare systems may benefit from auditing diagnostic delays and patterns of misdiagnosis across different phototypes. Standardized clinical photographic documentation may further support both clinical recognition and research in SOC populations.

The retrospective design of this study represents its main limitation, since it may have introduced selection bias and reduced the generalizability of the results. Nevertheless, our study adds to the limited body of evidence in this field and underscore the need for further research. Prospective multicenter studies and registry-based investigations including diverse skin phototypes will be essential to better define the epidemiological and clinical characteristics of dermatologic conditions in pediatric SOC patients.

## Figures and Tables

**Table 1:** Absolute and relative frequencies of various dermatological diagnoses between white and SOC populations. n.s.: not significant.

		White		SOC		*p*
AA	Median (IQR)	4.45 (1.62–8.85)		4.24 (1.32–8.15)		n.s.
		Absolute frequency	Relative frequency	Absolute frequency	Relative frequency	
Sex	Female	143	47.35	58	53.21	n.s.
	Male	159	52.65	51	46.79	
Diagnoses	1: Scabies 2: Entomodermatoses 3: Viral 4: Bacterial 5: Mycoses 6: Urticaria 7: Burns 8: Contact dermatitis 9: Atopic dermatitis (AD) 10: Seborrheic dermatitis 11: Purpura 12: Drug-related eruptions 13: Vascular lesions 14: Lichen planus 15: Acne 16: Psoriasis 17: Pruritus 18: Others	15 17 51 20 6 17 43 42 39 5 5 6 10 1 2 3 1 19	4.97 5.63 16.89 6.62 1.99 5.63 14.24 13.91 12.91 1.66 1.66 1.99 3.31 0.33 0.66 0.99 0.33 6.29	24 7 14 6 1 2 10 8 19 2 3 1 0 1 0 1 4 6	22.02 6.42 12.84 5.50 0.92 1.83 9.17 7.34 17.43 1.83 2.75 0.92 0.00 0.92 0.00 0.92 3.67 5.50	<0.001

**Table 2:** Demographic characteristics of SOC patients evaluated by a Pediatric Dermatologist for AD in 2019.

Total, n (%)	38 (100%)
Age class, n (%)	
- Infants and early childhood (<2 y)	9 (23.7%)
- Late childhood (2–12 y)	25 (65.8%)
- Adolescents (>12 y)	4 (10.5%)
Age (y), median (IQR)	5 (IQR 6.5)
Sex, n (%)	
- Female patients	21 (55.3%)
- Male patients	17 (44.7%)
Morphological variants of AD, n (%)	
- Lichenoid or follicular	12 (31.6%)
- Pityriasis alba	11 (28.9%)
- Prurigo nodularis	10 (26.3%)
- Classical	5 (13.2%)
Affected body locations, n *	
- Typical age-related sites	25
- Extensor surfaces	17

* Some patients had more than one affected area. y = years; IQR = interquartile range.

## Data Availability

The original contributions presented in this study are included in the article and supplementary material. Further inquiries can be directed to the corresponding author.

## Supplementary Materials

The following supporting information can be downloaded at: https://www.mdpi.com/article/10.3390/life16040578/s1. File S1: Additional methodological details (Classification of dermatological diagnoses used in this study and post hoc analysis).

## References

[1] Silverberg N.B., Durán-McKinster C., Tay Y.K. (2015). Pediatric Skin of Color.

[2] Perlman K.L., Williams N.M., Egbeto I.A., Gao D.X., Siddiquee N., Park J.H. (2021). Skin of color lacks representation in medical student resources: A cross-sectional study. Int. J. Womens Dermatol..

[3] Ahmed F., Maranga A., Lipoff J.B. (2022). Underrepresentation of skin of color in dermatology grand rounds cases: A single-center retrospective study. J. Am. Acad. Dermatol..

[4] Konisky H., Ortega K., Fayiga F., Balazic E., Kobets K., Kindred C. (2024). Underrepresentation of Skin of Color in Google Images Search of Common Skin, Hair, and Nail Conditions. J. Drugs Dermatol..

[5] Nutten S. (2015). Atopic Dermatitis: Global Epidemiology and Risk Factors. Ann. Nutr. Metab..

[6] Raimondo A., Lembo S. (2021). Atopic Dermatitis: Epidemiology and Clinical Phenotypes. Dermatol. Pract. Concept..

[7] Ricci G., Dondi A., Neri I., Ricci L., Patrizi A., Pession A. (2014). Atopic dermatitis phenotypes in childhood. Ital. J. Pediatr..

[8] Bylund S., von Kobyletzki L.B., Svalstedt M., Svensson Å. (2020). Prevalence and Incidence of Atopic Dermatitis: A Systematic Review. Acta Derm.-Venereol..

[9] Quan V.L., Erickson T., Daftary K., Chovatiya R. (2023). Atopic Dermatitis Across Shades of Skin. Am. J. Clin. Dermatol..

[10] Sachdeva M., Joseph M. (2022). Dermatology: How to manage atopic dermatitis in patients with skin of colour. Drugs Context.

[11] Neri I. (2022). Dermatite Atopica Del Lattante e Del Bambino.

[12] Noda S., Suárez-Fariñas M., Ungar B., Kim S.J., de Guzman Strong C., Xu H., Peng X., Estrada Y.D., Nakajima S., Honda T. (2015). The Asian atopic dermatitis phenotype combines features of atopic dermatitis and psoriasis with increased TH17 polarization. J. Allergy Clin. Immunol..

[13] Pippione M. (2015). Dermatologia e Malattie Sessualmente Trasmissibili.

[14] Gan C., Brand R., Foster R.S., Weidinger J., Rodrigues M. (2023). Diagnosis, assessment and management of atopic dermatitis in children with skin of colour. Aust. J. Gen. Pract..

[15] Davis C.M., Flohr C., Gupta M.R., Koplin J.J. (2023). Managing Atopic Dermatitis in Patients With Skin of Color. J. Allergy Clin. Immunol. Pract..

[16] Taylor S.C., Cook-Bolden F. (2002). Defining skin of color. Cutis.

[17] Monir R.L., Schoch J.J., Garvan C.W., Neu J., Lemas D.J. (2022). Association between atopic dermatitis and race from infancy to early childhood: A retrospective cohort study. Int. J. Dermatol..

[18] Spaziante M., Agresta A., D’Amato M., De Carli G., Tonziello G., Vantaggio V., Malatesta G.N., Girardi E., Barca A., Scognamiglio P. (2025). Post-COVID-19 resurgence of scabies’ cases in the Lazio Region, Italy: A new emerging public health threat?. Infect. Dis. Poverty.

[19] Honda T., Kabashima K. (2020). Reconciling innate and acquired immunity in atopic dermatitis. J. Allergy Clin. Immunol..

[20] Kramer O.N., Strom M.A., Ladizinski B., Lio P.A. (2017). The history of atopic dermatitis. Clin. Dermatol..

[21] Lopez Carrera Y.I., Al Hammadi A., Huang Y.H., Llamado L.J., Mahgoub E., Tallman A.M. (2019). Epidemiology, Diagnosis, and Treatment of Atopic Dermatitis in the Developing Countries of Asia, Africa, Latin America, and the Middle East: A Review. Dermatol. Ther..

[22] Nnoruka E.N. (2004). Current epidemiology of atopic dermatitis in south-eastern Nigeria. Int. J. Dermatol..

[23] Vachiramon V., Tey H.L., Thompson A.E., Yosipovitch G. (2012). Atopic Dermatitis in African American Children: Addressing Unmet Needs of a Common Disease. Pediatr. Dermatol..

[24] Herzum A., Occella C., Gariazzo L., Ciccarese G., Pastorino C., Matarese S., Marasini L., Viglizzo G. (2024). Clinical features of atopic dermatitis in pediatric patients with skin of color and comparison with different phototypes. Ski. Res. Technol..

[25] Cosickic A., Skokic F., Colic-Hadzic B., Jahic M. (2010). Clinical characteristics and estimation severity of the atopic dermatitis in children. Med. Arh..

[26] Hanifin J.M., Baghoomian W., Grinich E., Leshem Y.A., Jacobson M., Simpson E.L. (2022). The Eczema Area and Severity Index-A Practical Guide. Dermatitis.

[27] Faye O., Meledie N’Djong A.P., Diadie S., Coniquet S., Niamba P.A., Atadokpede F., Yoboue P.Y., Dieng M.T., Zkik A., Castagne C. (2020). Validation of the Patient-Oriented SCORing for Atopic Dermatitis tool for black skin. J. Eur. Acad. Dermatol. Venereol..

[28] Silverberg J.I., Gelfand J.M., Margolis D.J., Boguniewicz M., Fonacier L., Grayson M.H., Simpson E.L., Ong P.Y., Fuxench Z.C.C. (2018). Patient burden and quality of life in atopic dermatitis in US adults: A population-based cross-sectional study. Ann. Allergy Asthma Immunol..

[29] Fischer A.H., Shin D.B., Margolis D.J., Takeshita J. (2017). Racial and ethnic differences in healthcare utilization for childhood eczema: An analysis of the 2001–2013 Medical Expenditure Panel Surveys. J. Am. Acad. Dermatol..

[30] Kaufman B.P., Guttman-Yassky E., Alexis A.F. (2018). Atopic dermatitis in diverse racial and ethnic groups-Variations in epidemiology, genetics, clinical presentation and treatment. Exp. Dermatol..

[31] Brar K.K., Singh A.M., De Guzman N., Aquino M. (2023). Atopic Dermatitis: Diagnosis, Disparity, and Management in Children of Color. NASN Sch. Nurse.

[32] Boostani M., Bánvölgyi A., Zouboulis C.C., Goldfarb N., Suppa M., Goldust M., Lőrincz K., Kiss T., Nádudvari N., Holló P. (2025). Large language models in evaluating hidradenitis suppurativa from clinical images. J. Eur. Acad. Dermatol. Venereol..

[33] Boostani M., Bánvölgyi A., Goldust M., Cantisani C., Pietkiewicz P., Lőrincz K., Holló P., Wikonkál N.M., Paragh G., Kiss N. (2025). Diagnostic Performance of GPT-4o and Gemini Flash 2.0 in Acne and Rosacea. Int. J. Dermatol..

[34] Bellatreccia C., Zama D., Dondi A., Pierantoni L., Laura A., Neri I., Lanari M., Borghesi A., Calegari R. (2025). Addressing Bias and Data Scarcity in AI-Based Skin Disease Diagnosis with Non-Dermoscopic Images. Ceur Workshop Proceedings.

[35] Zama D., Borghesi A., Ranieri A., Manieri E., Pierantoni L., Andreozzi L., Dondi A., Neri I., Lanari M., Calegari R. (2024). Perspectives and Challenges of Telemedicine and Artificial Intelligence in Pediatric Dermatology. Children.

