# Supplementary material for: Skin of Color in Pediatric Dermatology: A Cross-Sectional Retrospective Analysis Addressing Inclusive Diagnosis and Care
Source: Life (Basel). 2026 Apr 1;16(4):578. doi: 10.3390/life16040578 (PMC13118273; doi:10.3390/life16040578)
Supplement: Supplementary file 1 [file life-16-00578-s001.zip › life-4170246-supplementary.pdf]

Classification of dermatological diagnoses used in this study

1. **Scabies**
2. **Entomodermatoses** (excluding scabies, e.g. prurigo strophulosum)

1. **Viral conditions** – including molluscum contagiosum, hand-foot-mouth disease, pityriasis rosea, herpes simplex, viral exanthems, Gianotti-Crosti syndrome, Asymmetric Periflexural Exanthem in Childhood (APEC)
2. **Bacterial infections** – including cold pyoderma, folliculitis, impetigo, abscesses, and others
3. **Mycoses**
4. **Urticaria**
5. **Burns**
6. **Contact dermatitis / diaper dermatitis** – including irritative and exogenous types
7. **Atopic dermatitis (AD)**
8. **Seborrheic dermatitis**
9. **Purpura**
10. **Vascular lesions**
11. **Other conditions** – including neonatal cephalic pustulosis, pilomatricoma, epidermal cyst, dyschromia, hyperkeratotic plaque, scalp nodules, dermatomyositis
12. **Psoriasis**
13. **Pruritus (unspecified)**
14. **Lichen planus / lichenoid eruptions**
15. **Acne**
16. **Drug-related eruptions / adverse drug reaction**

Post-hoc analysis

Group 1	Group 2	n	Adjusted p-value	Sig.
Scabies	Entomodermatoses	63	0.025	*
Scabies	Viral	104	< 0.001	*
Scabies	Bacterial	65	0.005	*
Scabies	Mycosis	46	0.0365	*
Scabies	Urticaria	58	< 0.001	*
Scabies	Burns	92	< 0.001	*
Scabies	Contact dermatitis	89	< 0.001	*
Scabies	AD	97	0.01	*

Scabies	Seborrheic dermatitis	46	n.s.	-
Scabies	Purpura	47	n.s.	-
Scabies	Drug-related eruptions	46	0.037	*
Scabies	Vascular lesions	49	< 0.001	*
Scabies	Lichen planus	41	n.s.	-
Scabies	Acne	41	n.s.	-
Scabies	Psoriasis	43	n.s.	-
Scabies	Pruritus	44	n.s.	-
Scabies	Other conditions	64	0.007	*
Entomodermatoses	Viral	89	n.s.	-
Entomodermatoses	Bacterial	50	n.s.	-
Entomodermatoses	Mycosis	31	n.s.	-
Entomodermatoses	Urticaria	43	n.s.	-
Entomodermatoses	Burns	77	n.s.	-
Entomodermatoses	Contact dermatitis	74	n.s.	-
Entomodermatoses	AD	82	n.s.	-
Entomodermatoses	Seborrheic dermatitis	31	n.s.	-
Entomodermatoses	Purpura	32	n.s.	-
Entomodermatoses	Drug-related eruptions	31	n.s.	-
Entomodermatoses	Vascular lesions	34	n.s.	-
Entomodermatoses	Lichen planus	26	n.s.	-
Entomodermatoses	Acne	26	n.s.	-
Entomodermatoses	Psoriasis	28	n.s.	-
Entomodermatoses	Pruritus	29	n.s.	-
Entomodermatoses	Other conditions	49	n.s.	-
Viral	Bacterial	91	n.s.	-
Viral	Mycosis	72	n.s.	-
Viral	Urticaria	84	n.s.	-
Viral	Burns	118	n.s.	-
Viral	Contact dermatitis	115	n.s.	-
Viral	AD	123	n.s.	-
Viral	Seborrheic dermatitis	72	n.s.	-
Viral	Purpura	73	n.s.	-
Viral	Drug-related eruptions	72	n.s.	-
Viral	Vascular lesions	75	n.s.	-
Viral	Lichen planus	67	n.s.	-
Viral	Acne	67	n.s.	-
Viral	Psoriasis	69	n.s.	-
Viral	Pruritus	70	0.014	*
Viral	Other conditions	90	n.s.	-

Bacterial	Mycosis	33	n.s.	-
Bacterial	Urticaria	45	n.s.	-
Bacterial	Burns	79	n.s.	-
Bacterial	Contact dermatitis	76	n.s.	-
Bacterial	AD	84	n.s.	-
Bacterial	Seborrheic dermatitis	33	n.s.	-
Bacterial	Purpura	34	n.s.	-
Bacterial	Drug-related eruptions	33	n.s.	-
Bacterial	Vascular lesions	36	n.s.	-
Bacterial	Lichen planus	28	n.s.	-
Bacterial	Acne	28	n.s.	-
Bacterial	Psoriasis	30	n.s.	-
Bacterial	Pruritus	31	0.027	*
Bacterial	Other conditions	51	n.s.	-
Mycosis	Urticaria	26	n.s.	-
Mycosis	Burns	60	n.s.	-
Mycosis	Contact dermatitis	57	n.s.	-
Mycosis	AD	65	n.s.	-
Mycosis	Seborrheic dermatitis	14	n.s.	-
Mycosis	Purpura	15	n.s.	-
Mycosis	Drug-related eruptions	14	n.s.	-
Mycosis	Vascular lesions	17	n.s.	-
Mycosis	Lichen planus	9	n.s.	-
Mycosis	Acne	9	n.s.	-
Mycosis	Psoriasis	11	n.s.	-
Mycosis	Pruritus	12	n.s.	-
Mycosis	Other conditions	32	n.s.	-
Urticaria	Burns	72	n.s.	-
Urticaria	Contact dermatitis	69	n.s.	-
Urticaria	AD	77	n.s.	-
Urticaria	Seborrheic dermatitis	26	n.s.	-
Urticaria	Purpura	27	n.s.	-
Urticaria	Drug-related eruptions	26	n.s.	-
Urticaria	Vascular lesions	29	n.s.	-
Urticaria	Lichen planus	21	n.s.	-
Urticaria	Acne	21	n.s.	-
Urticaria	Psoriasis	23	n.s.	-
Urticaria	Pruritus	24	0.006	*
Urticaria	Other conditions	44	n.s.	-
Burns	Contact dermatitis	103	n.s.	-
Burns	DA	111	n.s.	-

Burns	Seborrheic dermatitis	60	n.s.	-
Burns	Purpura	61	n.s.	-
Burns	Drug-related eruptions	60	n.s.	-
Burns	Vascular lesions	63	n.s.	-
Burns	Lichen planus	55	n.s.	-
Burns	Acne	55	n.s.	-
Burns	Psoriasis	57	n.s.	-
Burns	Pruritus	58	0.010	*
Burns	Other conditions	78	n.s.	-
Contact dermatitis	AD	108	n.s.	-
Contact dermatitis	Seborrheic dermatitis	57	n.s.	-
Contact dermatitis	Purpura	58	n.s.	-
Contact dermatitis	Drug-related eruptions	57	n.s.	-
Contact dermatitis	Vascular lesions	60	n.s.	-
Contact dermatitis	Lichen planus	52	n.s.	-
Contact dermatitis	Acne	52	n.s.	-
Contact dermatitis	Psoriasis	54	n.s.	-
Contact dermatitis	Pruritus	55	0.006	*
Contact dermatitis	Other conditions	75	n.s.	-
AD	Seborrheic dermatitis	65	n.s.	-
AD	Purpura	66	n.s.	-
AD	Drug-related eruptions	65	n.s.	-
AD	Vascular lesions	68	n.s.	-
AD	Lichen planus	60	n.s.	-
AD	Acne	60	n.s.	-
AD	Psoriasis	62	n.s.	-
AD	Pruritus	63	n.s.	-
AD	Other conditions	83	n.s.	-
Seborrheic dermatitis	Purpura	15	n.s.	-
Seborrheic dermatitis	Drug-related eruptions	14	n.s.	-
Seborrheic dermatitis	Vascular lesions	17	n.s.	-
Seborrheic dermatitis	Lichen planus	9	n.s.	-
Seborrheic dermatitis	Acne	9	n.s.	-
Seborrheic dermatitis	Psoriasis	11	n.s.	-
Seborrheic dermatitis	Pruritus	12	n.s.	-

Seborrheic dermatitis	Other conditions	32	n.s.	-
Porpora	Drug-related eruptions	15	n.s.	-
Porpora	Vascular lesions	18	n.s.	-
Porpora	Lichen planus	10	n.s.	-
Porpora	Acne	10	n.s.	-
Porpora	Psoriasis	12	n.s.	-
Porpora	Pruritus	13	n.s.	-
Porpora	Other conditions	33	n.s.	-
Reazione a farmaci	Vascular lesions	17	n.s.	-
Reazione a farmaci	Lichen planus	9	n.s.	-
Reazione a farmaci	Acne	9	n.s.	-
Reazione a farmaci	Psoriasis	11	n.s.	-
Reazione a farmaci	Pruritus	12	n.s.	-
Reazione a farmaci	Other conditions	32	n.s.	-
Vascular lesion	Lichen planus	12	n.s.	-
Vascular lesion	Acne	12	n.s.	-
Vascular lesion	Psoriasis	14	n.s.	-
Vascular lesion	Pruritus	15	0.004	*
Vascular lesion	Other conditions	35	n.s.	-
Lichen planus	Acne	4	n.s.	-
Lichen planus	Psoriasis	6	n.s.	-
Lichen planus	Pruritus	7	n.s.	-
Lichen planus	Other conditions	27	n.s.	-
Acne	Psoriasis	6	n.s.	-
Acne	Pruritus	7	n.s.	-
Acne	Other conditions	27	n.s.	-
Psoriasis	Pruritus	9	n.s.	-
Psoriasis	Other conditions	29	n.s.	-
Pruritus	Other conditions	30	0.031	*
